# Supplementary material for: Does Measles, Mumps, and Rubella (MMR) Vaccination Protect against COVID-19 Outcomes: A Nationwide Cohort Study
Source: Vaccines (Basel). 2022 Nov 16;10(11):1938. doi: 10.3390/vaccines10111938 (PMC9694832; doi:10.3390/vaccines10111938)
Supplement: Supplementary file 1 [file vaccines-10-01938-s001.zip › vaccines-2014439-supplementary.pdf]

Supplementary Materials

Table of Contents

Supplementary methods 2
Codes used to define MMR vaccine exposure 2
Categories of complex chronic conditions and the corresponding ICD-10 Diagnosis 4
Supplementary Tables 7
Table S1. Analysis of the association between MMR vaccine exposure and hospitalization for Covid-19 7
Table S2. Sensitivity analysis of the association between exposure to MMR vaccine and the risk of hospitalization for Covid-19 9
Table S3. Analysis of the association between MMR vaccine exposure and hospitalization with Covid-19 10
Table S4. Analysis of the association between MMR vaccine exposure and hospitalization with Covid-19 12
Table S5. Analysis of the association between MMR vaccine exposure and hospitalization with Covid-19 14
Supplementary figures 16
Figure S1. Standardized mean differences before and after inverse probability of weighting 16

Supplementary methods

Codes used to define MMR vaccine exposure

CIP13	label	ATC	Status
3400932777655	R.O.R., poudre et solvant pour solution injectable. vaccin à virus vivants atténués contre la rougeole, les oreillons et la rubéole	J07BD52	Archived
3400933673567	IMMU ORR, poudre et solvant pour suspension injectable. Vaccin rougeoleux, des oreillons et rubéoleux, atténué.	J07BD52	Archived
3400933673628	R.O.R. VAX, poudre et solvant pour suspension injectable. Vaccin rougeoleux, des oreillons et rubéoleux atténué.	J07BD52	Archived
3400935137340	PRIORIX, poudre et solvant pour solution injectable en seringue préremplie. Vaccin rougeoleux, des oreillons et rubéoleux vivant	J07BD52	Active
3400937327848	M-M-RVAXPRO, poudre et solvant pour suspension injectable. Vaccin rougeoleux, des oreillons, et rubéoleux (vivant)	J07BD52	Active
3400937328210	M-M-RVAXPRO poudre et solvant pour suspension injectable en seringue préremplie.Vaccin rougeoleux, des oreillons, et rubéoleux (vivant).	J07BD52	Active
3400931698401	D T BIS RUDIVAX, vaccin	J07BD52	Archived
3400932777716	R.O.R., poudre et solvant pour solution injectable. vaccin à virus vivants atténués contre la rougeole, les oreillons et la rubéole	J07BD52	Archived
3400932777884	R.O.R., poudre et solvant pour solution injectable. vaccin à virus vivants atténués contre la rougeole, les oreillons et la rubéole	J07BD52	Archived
3400933158569	TRIMOVAX PASTEUR, poudre et solvant pour solution injectable. vaccin à virus vivants atténués contre la rougeole, les oreillons et la rubéole	J07BD52	Archived
3400933158620	TRIMOVAX PASTEUR, poudre et solvant pour solution injectable. vaccin à virus vivants atténués contre la rougeole, les oreillons et la rubéole	J07BD52	Archived
3400933158798	TRIMOVAX PASTEUR, poudre et solvant pour solution injectable. vaccin à virus vivants atténués contre la rougeole, les oreillons et la rubéole	J07BD52	Archived
3400933647605	IMMU ORR, poudre et solvant pour suspension injectable. Vaccin rougeoleux, des oreillons et rubéoleux, atténué.	J07BD52	Archived
3400935081308	PRIORIX, poudre et solvant pour solution injectable en seringue préremplie. Vaccin rougeoleux, des oreillons et rubéoleux vivant	J07BD52	Active
3400935081476	PRIORIX, poudre et solvant pour solution injectable en seringue préremplie. Vaccin rougeoleux, des oreillons et rubéoleux vivant	J07BD52	Active
3400935081537	PRIORIX, poudre et solvant pour solution injectable en seringue préremplie. Vaccin rougeoleux, des oreillons et rubéoleux vivant	J07BD52	Active
3400935081766	PRIORIX, poudre et solvant pour solution injectable en seringue préremplie. Vaccin rougeoleux, des oreillons et rubéoleux vivant	J07BD52	Active
3400935136978	PRIORIX, poudre et solvant pour solution injectable en seringue préremplie. Vaccin rougeoleux, des oreillons et rubéoleux vivant	J07BD52	Active
3400935137050	PRIORIX, poudre et solvant pour solution injectable en seringue préremplie. Vaccin rougeoleux, des oreillons et rubéoleux vivant	J07BD52	Active

3400935137111	PRIORIX, poudre et solvant pour solution injectable en seringue préremplie. Vaccin rougeoleux, des oreillons et rubéoleux vivant	J07BD52	Active
3400935137289	PRIORIX, poudre et solvant pour solution injectable en seringue préremplie. Vaccin rougeoleux, des oreillons et rubéoleux vivant	J07BD52	Active
3400935137401	PRIORIX, poudre et solvant pour solution injectable en seringue préremplie. Vaccin rougeoleux, des oreillons et rubéoleux vivant	J07BD52	Active
3400935137579	PRIORIX, poudre et solvant pour solution injectable en seringue préremplie. Vaccin rougeoleux, des oreillons et rubéoleux vivant	J07BD52	Active
3400935137630	PRIORIX, poudre et solvant pour solution injectable en seringue préremplie. Vaccin rougeoleux, des oreillons et rubéoleux vivant	J07BD52	Active
3400935965134	IMMU ORR, poudre et solvant pour suspension injectable. Vaccin rougeoleux, des oreillons et rubéoleux, atténué.	J07BD52	Archived
3400935965363	R.O.R. VAX, poudre et solvant pour suspension injectable. Vaccin rougeoleux, des oreillons et rubéoleux atténué.	J07BD52	Archived
3400937084840	R.O.R., poudre et solvant pour solution injectable. vaccin à virus vivants atténués contre la rougeole, les oreillons et la rubéole	J07BD52	Archived
3400937327909	M-M-RVAXPRO poudre et solvant pour suspension injectable en seringue préremplie.Vaccin rougeoleux, des oreillons, et rubéoleux (vivant).	J07BD52	Active
3400937328098	M-M-RVAXPRO poudre et solvant pour suspension injectable en seringue préremplie.Vaccin rougeoleux, des oreillons, et rubéoleux (vivant).	J07BD52	Active
3400937328159	M-M-RVAXPRO poudre et solvant pour suspension injectable en seringue préremplie.Vaccin rougeoleux, des oreillons, et rubéoleux (vivant).	J07BD52	Active
3400939211206	R.O.R., poudre et solvant pour solution injectable. vaccin à virus vivants atténués contre la rougeole, les oreillons et la rubéole	J07BD52	Archived
3400956702602	M-M-RVAXPRO poudre et solvant pour suspension injectable en seringue préremplie.Vaccin rougeoleux, des oreillons, et rubéoleux (vivant).	J07BD52	Active
3400956702770	M-M-RVAXPRO poudre et solvant pour suspension injectable en seringue préremplie.Vaccin rougeoleux, des oreillons, et rubéoleux (vivant).	J07BD52	Active
3400956702831	M-M-RVAXPRO poudre et solvant pour suspension injectable en seringue préremplie.Vaccin rougeoleux, des oreillons, et rubéoleux (vivant).	J07BD52	Active
3400956703081	M-M-RVAXPRO poudre et solvant pour suspension injectable en seringue préremplie.Vaccin rougeoleux, des oreillons, et rubéoleux (vivant).	J07BD52	Active
3400956703142	M-M-RVAXPRO poudre et solvant pour suspension injectable en seringue préremplie.Vaccin rougeoleux, des oreillons, et rubéoleux (vivant).	J07BD52	Active
3400956722181	M-M-RVAXPRO, poudre et solvant pour suspension injectable. Vaccin rougeoleux, des oreillons, et rubéoleux (vivant)	J07BD52	Active
3400956722242	M-M-RVAXPRO poudre et solvant pour suspension injectable en seringue préremplie.Vaccin rougeoleux, des oreillons, et rubéoleux (vivant).	J07BD52	Active
3400956722303	M-M-RVAXPRO poudre et solvant pour suspension injectable en seringue préremplie.Vaccin rougeoleux, des oreillons, et rubéoleux (vivant).	J07BD52	Active

Categories of complex chronic conditions and the corresponding ICD-10 Diagnosis

Categories	Subcategories	International Classification of Diseases (ICD-10)<br>Long-term Conditions (ALD)<br>Groupe Homogène de Malade (GHM)<br>Common Classification of Medical Acts (CCAM)	Definition
**Neurological and Neuromuscular**	Brain and spinal cord malformations	Q00-Q07, G90.1	ICD-10 diagnosis of hospitalization in the previous 5 years or long-term condition (ALD) in 2019
	CNS degeneration and diseases	E75.0, E75.1, E75.2, E75.4, F84.2, G11.1-G11.4, G11.8, G11.9, G12.0-G12.2, G12.8, G12.9, G31.0, G31.8, G31.9, G32.8, G93.8, G93.9, G94, G91.1, G25.3, G95.1, G95.8, G90.9, Q85.1	
	Infantile cerebral palsy	G80	
	Other disorders of CNS	G37.1, G37.2, G37.8, G81.9, G82.5, G83.9, G93.1, G93.5, R40.20	
	Occlusion of cerebral arteries	I63.3, I63.5	
	Muscular dystrophies and myopathies	G71, G72	
	Movement diseases	G10, G20, G21.0, G21.1, G21.8, G23.0-G23.2, G23.8, G24.0, G24.8, G25.3-G25.5, G25.8, G25.9, G80.3	
	Epilepsy	G40, G41	
		Antiepileptic drug	At least 3 antiepileptic drug deliveries in 2019
	Devices	T85.1, T85.7, Z98.2, Z45.84	ICD-10 diagnosis of hospitalization in the previous 5 years
**Psychiatric diseases**	Non-organic psychosis	F29	ICD-10 diagnosis of hospitalization in the previous 5 years or long-term condition (ALD) in 2019
	Depression	F32	
	Anxiety disorders	F41	
	Personality disorder (Dependent personality, Anxious (avoidant) personality, Anankastic personality, Histrionic personality, Emotionally labile personality, Dissocial Personality, Schizoid Personality, Paranoid Personality)	F60	
	Mental retardation	F70 to F73, F78, F79	
	Developmental disability (Autism, Hyperactivity associated with mental retardation and stereotyped movements, Other disorder, Asperger's Syndrome, Rett syndrome)	F80 to F84	
	Mixed conduct and emotional disorders	F92	
**Cardiovascular**	Heart and great vessel malformations	Q20, Q21-Q24, Q25.1/9-Q26, Q28.2, Q28.3, Q28.9	

Categories	Subcategories	International Classification of Diseases (ICD-10)<br>Long-term Conditions (ALD)<br>Groupe Homogène de Malade (GHM)<br>Common Classification of Medical Acts (CCAM)	Definition
	Endocardium diseases	I34.0, I34.8, I36.0, I36.8, I37.0, I37.8	ICD-10 diagnosis of hospitalization in the previous 5 years or long-term condition (ALD) in 2019
	Cardiomyopathies	I42, I43, I51.5	
	Conduction disorder	I44, I45, I47, I48, I49.0	
	Dysrhythmias	I49.1-I49.5, I49.8, I49.9, R00.1	
	Other	I27.0, I27.1, I27.2, I27.8, I27.9, I50.9, I51.7, I63.1, I63.2, Z95.1	
	Devices	T82.5, T82.110A, T82.1, T82.0, T82.2, T82.6, T82.7, Z95.0, Z95.2, Z95.3, Z95.8, Z95.8, Z45.0, Z95.9	ICD-10 diagnosis of hospitalization in the previous 5 years
**Respiratory**	Respiratory malformations	Q30-Q34, P28.0	ICD-10 diagnosis of hospitalization in the previous 5 years or long-term condition (ALD) in 2019
	Chronic respiratory diseases	J40-J47, J96.1, J84.1, Z90.2, J98	
	Cystic fibrosis	E84	
	Devices	J95.0, Z43.0, Z93.0, Z99.0, Z99.1	
	Drug	ATC code starting by R03	At least 2 specific drugs were used in 2019
**Renal and Urological**	Congenital anomalies	Q60-Q64	ICD-10 diagnosis of hospitalization in the previous 5 years or long-term condition (ALD) in 2019
	Chronic renal failure	N18	
	Other	Z90.5, Z90.6	
	Chronic bladder diseases	G83.4, N31.2, N31.9	
	Devices	T85.7, Z93.5, Z93.6, Z91.1, Z99.2, Z43.5, Z43.6, Z46.6	
**Gastrointestinal**	Congenital anomalies	Q39.0-Q39.4, Q41-Q45	ICD-10 diagnosis of hospitalization in the previous 5 years or long-term condition (ALD) in 2019
	Chronic liver disease and cirrhosis	K73, K74, K75.4, K76.0-K76.3, K76.5, K76.8	
	Inflammatory bowel diseases	K50, K51	
	Other	I82.0, K55.1, K56.2, K59.3, Z98.0, Z90.3, Z90.49	
	Devices	Z93.1-Z93.4, Z43.1-Z43.4	
**Hematological or immunological**	Hereditary anemias	D55-D58	ICD-10 diagnosis of hospitalization in the previous 5 years or long-term condition (ALD) in 2019
	Aplastic anemias	D60-D61, D71	
	Hereditary immunodeficiency	D80-D89, D72.0, M30.3, M35.9	
	Coagulation/hemorrhagic	D66, D68.2, D69.41, D69.4	
	Leukopenia	D70	
	Hemophagocytic Syndromes	D76.1-D76.3	

Categories	Subcategories	**International Classification of Diseases (ICD-10)**<br>**Long-term Conditions (ALD)**<br>**Groupe Homogène de Malade (GHM)**<br>**Common Classification of Medical Acts (CCAM)**	Definition
	Sarcoidosis	D86.9	
	Acquired immunodeficiency	B20-B24	
	Polyarteritis nodosa and related conditions	M30.0, M31.0, M31.1, M31.3, M31.4, M31.6	
	Diffuse diseases of connective tissue	M32.1, M33.9, M34.0, M34.1, M34.9	
**Metabolic**	Amino acid metabolism	E70.0, E70.2, E70.3, E70.8, E71.0-E71.3, E72.0-E72.4, E72.8, E72.9	ICD-10 diagnosis of hospitalization in the previous 5 years or long-term condition (ALD) in 2019
	Carbohydrate metabolism	E74.0-E74.4, E74.8, E74.9	
	Lipid metabolism	E75, E77.0, E77.1, E78.0-E78.4, E78.5-E78.9, E88.1, E88.8	
	Storage disorder	E76.0-E76.3, E85	
	Other metabolic disorders	E79.1, E79.8, E80.4-E80.7, E83.0, E83.1, E83.3, E83.4, D84.1, E88, H49.8	
	Endocrine disorders	E00.9, E23.0, E23.2, E22.2, E23.3, E23.7, E24.0, E24.2, E24.3, E24.8, E24.9, E26.8, E25.0, E25.8, E25.9	
	Diabetes	E10-E14	ICD-10 diagnosis of hospitalization in the previous 2 years or long-term condition (ALD) in 2019
		Diabetes drug: Insulin	At least 3 insulin drugs in the previous 2 years
**Other Congenital or Genetic Defects**	Chromosomal anomalies	Q90, Q91.3, Q91.4, Q91.7, Q92.8, Q93, Q95.0, Q96.9, Q97, Q98, Q99.8, Q99.9	ICD-10 diagnosis of hospitalization in the previous 5 years or long-term condition (ALD) in 2019
	Bone and joint anomalies	E34.3, M41.0, M41.2, M41.30, M41.8, M41.9, M43.3, M96.5, Q72.2, Q75.0, Q75.2, Q75.9, Q76.0-Q76.2, Q76.4-Q76.7, Q77, Q78.0-Q78.4, Q78.8, Q78.9	
	Diaphragm and abdominal wall	K44.9, Q79.0-Q79.5, Q79.9	
	Other congenital anomalies	Q81, Q87.1-Q87.3, Q87.4, Q87.8, Q89.7, Q89.9, Q99.2	
**Cancer**	Neoplasms	C00-C96, D01-D09, D37-D49, Q85.0	ICD-10 diagnosis of hospitalization in the previous 2 years

Supplementary Tables

Table S1. Analysis of the association between MMR vaccine exposure and hospitalization for Covid-19

	Main diagnosis of Covid-19			
	**Yes (N = 911)**	**No (N = 7,183,793)**	**Crude OR [IC95%]**	**Adjusted OR aOR [IC95%]**
**Exposed to MMR vaccine**	**873 (95.8)**	**6,799,669 (94.6)**	**1.29 [0.93-1.79]**	**1.19 [0.85-1.65]**
**Age (years) - Median [IIQ]**	5 [2-8]	6 [3-8]	0.94 [0.92-0.97]	
**Age (years) – n (%)**				
1-4	397 (43.6)	2,636,277 (36.7)	1	1
5-9	399 (43.8)	3,632,231 (50.6)	0.72 [0.63-0.83]	1.03 [0.88-1.20]
10-12	115 (12.6)	915,285 (12.7)	0.83 [0.67-1.02]	1.29 [1.03-1.61]
**Sex – n (%)**				
Male	501 (55.0)	3,678,207 (51.2)	1	1
Female	410 (45.0)	3,505,586 (48.8)	0.85 [0.75-0.97]	0.94 [0.83-1.07]
**CMU beneficiaries – n (%)**	292 (32.0)	1,142,727 (15.9)	2.49 [2.17-2.86]	1.90 [1.64-2.19]
**Region – n (%)**				
Île-de-France	254 (27.9)	1,413,459 (19.7)	1	1
Northwest	94 (10.3)	1,444,186 (20.1)	0.36 [0.28-0.45]	0.31 [0.24-0.40]
Northeast	234 (25.7)	1,591,835 (22.2)	0.81 [0.68-0.97]	0.60 [0.49-0.73]
Southeast	264 (29.0)	1,804,777 (25.1)	0.81 [0.68-0.96]	0.71 [0.59-0.85]
Southwest	62 (6.8)	905,380 (12.6)	0.38 [0.28-0.50]	0.32 [0.24-0.42]
Unknown	3 (0.3)	24,156 (0.3)		
**Social deprivation index (quintiles) – n (%)**				
1 (the least deprivation)	147 (16.1)	1,434,948 (20.0)	1	1
2	167 (18.3)	1,452,780 (20.2)	1.12 [0.89-1.14]	1.33 [1.06-1.68]
3	148 (16.3)	1,401,248 (19.5)	1.03 [0.82-1.29]	1.23 [0.97-1.56]
4	185 (20.3)	1,369,610 (19.1)	1.31 [1.06-1.63]	1.55 [1.23-1.96]

5 (the most deprivation)	257 (28.2)	1,392,926 (19.4)	1.80 [1.47-2.10]		1.81 [1.45-2.26]
Unknown	7 (0.8)	132,281 (1.8)			
**Exposed to other vaccine – n (%)**	902 (99.0)	7,066,036 (98.4)	1.67 [0.86-3.20]		
**Number of previous consultations with a general practitioner or pediatrician– n (%)**					
No consultation	53 (5.8)	559,322 (7.9)	1.07 [0.78-1.73]		1.07 [0.78-1.48]
1-3	128 (14.0)	1,451,189 (20.2)	1		1
4-7	202 (22.2)	2,000,077 (27.8)	1.14 [0.91-1.42]		1.07 [0.86-1.34]
8-12	191 (21.0)	1,560,487 (21.7)	1.38 [1.10-1.73]		1.12 [0.89-1.41]
13 or more	337 (37.0)	1,612,718 (22.4)	2.36 [1.93-2.90]		1.39 [1.10-1.74]
**Previous hospitalizations – n (%)**	212 (23.3)	329,962 (4.6)	6.29 [5.40-7.34]		3.91 [3.30-4.63]
**Comorbidities – n (%)**					
Neurological/Neuromuscular	56 (6.1)	55,676 (0.8)	8.38 [6.39-10.9]		
Respiratory	180 (19.7)	581,004 (8.1)	2.79 [2.37-3.29]		
Cardiovascular	18 (2.0)	21,783 (0.3)	6.62 [4.15-10.5]		
Psychiatric	29 (3.2)	92,206 (1.3)	2.52 [1.74-3.66]	**At least one comorbidities**	2.73 [2.34-3.18]
Metabolic	18 (2.0)	14,679 (0.2)	9.86 [6.18-15.7]		
Gastrointestinal	13 (1.4)	8,684 (0.1)	11.9 [6.91-20.6]		
Renal/Urological	10 (1.1)	13,514 (0.2)	5.90 [3.16-11.0]		
Hematological/Immunological	51 (5.6)	15,222 (0.2)	27.9 [21.0-37.0]		
Genetic/Congenital defect	19 (2.1)	20,333 (0.3)	7.50 [4.76-11.8]		
Cancer	13 (1.4)	5,295 (0.1)	19.6 [11.3-33.9]		
**Intensive Care Unit Admission / death**	73/1				

Table S2. Sensitivity analysis of the association between exposure to MMR vaccine and the risk of hospitalization for Covid-19

	No of events/no of individuals	aOR [95%CI]*
**Sensitivity analysis**		
**Exposed to the first or second dose of MMR vaccine one year before the index date (15 February 2020)**	366/2,201,114	1.21 [0.91-1.62]
**Exposed to the first or second dose of MMR vaccine two year before the index date (15 February 2020)**	441/2,853,425	1.13 [0.85-1.51]

* Covariates used for inverse probability of weighting: age, sex, CMU, region of residence, social deprivation index, previous consultations, previous hospitalizations, and comorbidities.

Table S3. Analysis of the association between MMR vaccine exposure and hospitalization with Covid-19

	Main diagnosis of Covid-19 and PIMS			
	**Yes (N = 1,580)**	**No (N = 7,183,124)**	**Crude OR [IC95%]**	**Adjusted OR aOR [IC95%]**
**Exposed to MMR vaccine**	**1,507 (95,4)**	**6,799,035 (94.6)**	**1.16 [0.92-1.47]**	**1.05 [0.83-1.34]**
**Age (years) - Median [IIQ]**	5 [2-8]	6 [3-8]		
**Age (years) – n (%)**				
1-4	782 (49.5)	2,635,892 (36,7)	1	1
5-9	642 (40.6)	3,631,988 (50.6)	0.59 [0.53-0.66]	0.78 [0.69-0.87]
10-12	156 (9.9)	915,244 (12.7)	0.57 [0.48-0.68]	0.80 [0.67-0.96]
**Sex – n (%)**				
Male	901 (57.0)	3,677,807 (51,2)	1	1
Female	679 (43.0)	3,505,317 (48,8)	0.79 [0.71-0.87]	0.85 [0.77-0.94]
**CMU beneficiaries – n (%)**	431 (27.3)	1,142,588 (15.9)	1.98 [1.77-2.21]	1.61 [1.43-1.81]
**Region – n (%)**				
Île-de-France	492 (31.1)	1,413,221 (19.7)	1	1
Northwest	179 (11.3)	1,444,101 (20.1)	0.35 [0.30-0.42]	0.32 [0.27-0.38]
Northeast	354 (22.4)	1,591,715 (22.2)	0.63 [0.55-0.73]	0.51 [0.44-0.59]
Southeast	425 (26.9)	1,804,616 (25.1)	0.67 [0.59-0.77]	0.62 [0.54-0.71]
Southwest	126 (8.0)	905,316 (12.6)	0.40 [0.32-0.48]	0.35 [0.29-0.43]
Unknown	4 (0.3)	24,155 (0.3)		
**Social deprivation index (quintiles) – n (%)**				
1 (the least deprivation)	302 (19.1)	1,434,793 (20.0)	1	1
2	303 (19.2)	1,452,644 (20.2)	0.99 [0.84-1.16]	1.23 [1.04-1.45]
3	269 (17.0)	1,401,127 (19.5)	0.91 [0.77-1.07]	1.17 [0.98-1.39]
4	290 (18.3)	1,369,505 (19.1)	1.01 [0.85-1.18]	1.29 [1.09-1.53]
5 (the most deprivation)	399 (25.2)	1,392,784 (19.4)	1.36 [1.17-1.58]	1.55 [1.32-1.83]
Unknown	17 (1.2)	132,271 (1.8)		

**Exposed to other vaccine – n (%)**	1,559 (98.6)	7,065,379 (98.4)	1.23 [0.80-1.90]		
**Number of previous consultations with a general practitioner or pediatrician– n (%)**					
No consultation	96 (6.0)	559,279 (7.8)	1.15 [0.90-1.46]		1.13 [0.89-1.44]
1-3	216 (13.7)	1,451,101 (20.2)	1		1
4-7	371 (23.5)	1,999,908 (27.8)	1.24 [1.05-1.45]		1.16 [0.98-1.38]
8-12	325 (20.6)	1,560,353 (21.8)	1.39 [1.17-1.66]		1.11 [0.93-1.32]
13 or more	572 (36.2)	1,612,483 (22.4)	2.38 [2.03-2.78]		1.39 [1.16-1.65]
**Previous hospitalizations – n (%)**	300 (18.9)	329,874 (4.6)	4.87 [4.29-5.52]		3.08 [2.68-3.53]
**Comorbidities – n (%)**					
Neurological/Neuromuscular	66 (4.2)	55,666 (0.8)	5.58 [4.36-7.14]		
Respiratory	268 (17.0)	580,916 (8.1)	2.32 [2.03-2.64]		
Cardiovascular	32 (2.0)	21,786 (0.3)	6.80 [4.79-9.65]		
Psychiatric	36 (2.3)	92,199 (1.3)	1.79 [1.28-2.49]	**At least one comorbidities**	2.23 [1.98-2.52]
Metabolic	20 (1.3)	14,677 (0.2)	6.26 [4.02-9.73]		
Gastrointestinal	17 (1.1)	8,680 (0.1)	8.99 [5.57-14.5]		
Renal/Urological	11 (0.7)	13,513 (0.2)	3.72[2.05-6.73]		
Hematological/Immunological	79 (5.0)	15,194 (0.2)	24.8 [19.7-31.1]		
Genetic/Congenital defect	21 (1.3)	20,331 (0.3)	4.74[3.08-7.30]		
Cancer	15 (0.9)	5,293 (0.1)	13.0[9.81-21.6]		
**Intensive Care Unit Admission / death**	103/1				

Table S4. Analysis of the association between MMR vaccine exposure and hospitalization with Covid-19

	Main and associated diagnosis of Covid-19				
	**Yes (N = 1,830)**	**No (N = 7,182,874)**	**Crude OR [IC95%]**	**Adjusted OR aOR [IC95%]**	**aOR [IC95%] (IPTW)**
**Exposed to MMR vaccine**	1,747 (95.5)	6,798,795 (94.6)	1.18 [0.95-1.48]	1.04 [0.83-1.30]	0.91 [0.75-1.11]
**Age (years) - Median [IIQ]**	5 [2-8]	6 [3-8]			
**Age (years) – n (%)**					
1-4	898 (49.1)	2,635,776 (36.7)	1	1	
5-9	739 (40.4)	3,631,891 (50.6)	0.59 [0.54-0.65]	0.85 [0.77-0.95]	
10-12	193 (10.5)	915,207 (12.7)	0.61 [0.53-0.72]	0.96 [0.82-1.14]	
**Sex – n (%)**					
Male	1,032 (56.4)	3,677,676 (51.2)	1	1	
Female	798 (43.6)	3,505,198 (48.8)	0.81 [0.74-0.89]	0.90 [0.82-0.99]	
**CMU beneficiaries – n (%)**	550 (30.0)	1,142,469 (15.9)	2.27 [2.05-2.51]	1.82 [1.64-2.03]	
**Region – n (%)**					
Île-de-France	764 (41.7)	1,412,949 (19.7)	1	1	
Northwest	143 (7.8)	1,444,137 (20.1)	0.18 [0.15-0.21]	0.17 [0.14-0.20]	
Northeast	396 (21.6)	1,591,673 (22.2)	0.46 [0.40-0.52]	0.37 [0.32-0.42]	
Southeast	408 (22.4)	1,804,633 (25.1)	0.41 [0.37-0.47]	0.38 [0.34-0.43]	
Southwest	116 (6.3)	905,326 (12.6)	0.23 [0.19-0.28]	0.21 [0.17-0.25]	
Unknown	3 (0.2)	24,156 (0.3)			
**Social deprivation index (quintiles) – n (%)**					
1 (the least deprivation)	423 (23.1)	1,434,672 (20.0)	1	1	
2	325 (17.8)	1,452,622 (20.2)	0.75 [0.65-0.87]	1.06 [0.92-1.24]	
3	301 (16.4)	1,401,095 (19.5)	0.72 [0.62-0.84]	1.13 [0.96-1.32]	
4	323 (17.6)	1,369,472 (19.1)	0.80 [0.69-0.92]	1.22 [1.04-1.42]	
5 (the most deprivation)	442 (24.1)	1,392,741 (19.4)	1.07 [0.94-1.23]	1.37 [1.18-1.58]	
Unknown	16 (1.0)	132,272 (1.8)			

**Exposed to other vaccine – n (%)**	1,796 (98.1)	7,065,142 (98.4)	0.88[0.62-1.23]		
**Number of previous consultations with a general practitioner or pediatrician– n (%)**					
No consultation	97 (5.3)	559,278 (7.9)	1.00 [0.79-1.26]		1.01 [0.80-1.28]
1-3	251 (13.7)	1,451,066 (20.2)	1		1
4-7	396 (21.7)	1,999,883 (27.8)	1.14 [0.97-1.34]		1.05 [0.90-1.24]
8-12	387 (21.1)	1,560,291 (21.7)	1.43 [1.22-1.68]		1.09 [0.92-1.28]
13 or more	699 (38.2)	1,612,356 (22.4)	2.50 [2.17-2.89]		1.31 [1.12-1.55]
**Previous hospitalizations – n (%)**	453 (24.7)	329,721 (4.6)	6.83 [6.14-7.60]		3.85 [3.42-4.32]
**Comorbidities – n (%)**					
Neurological/Neuromuscular	125 (6.8)	55,607 (0.8)	9.40 [7.83-11.2]		
Respiratory	433 (23.6)	580,751 (8.1)	3.52 [3.16-3.92]		
Cardiovascular	41 (2.2)	21,760 (0.3)	7.54 [5.53-10.2]		
Psychiatric	67 (3.6)	92,168 (1.3)	2.92 [2.29-3.73]	**At least one comorbidities**	3.27 [2.94-3.63]
Metabolic	39 (2.1)	14,658 (0.2)	10.6 [7.75-14.6]		
Gastrointestinal	28 (1.5)	8,669 (0.1)	12.8 [8.85-18.6]		
Renal/Urological	21 (1.1)	13,503 (0.2)	6.16 [4.01-9.48]		
Hematological/Immunological	93 (5.0)	15,180 (0.2)	25.2 [20.5-31.1]		
Genetic/Congenital defect	52 (2.8)	20,300 (0.3)	10.3 [7.83-13.6]		
Cancer	24 (1.3)	5,284 (0.1)	18.1 [12.1-27.0]		
**Intensive Care Unit Admission / death**	132/5				

Table S5. Analysis of the association between MMR vaccine exposure and hospitalization with Covid-19

	Main/associated diagnosis of Covid-19 and PIMS				
	**Yes (N = 2,418)**	**No (N = 7,182,286)**	**Crude OR [IC95%]**	**Adjusted OR aOR [IC95%]**	**OR [IC95%] (IPTW)**
**Exposed to MMR vaccine**	2,305 (95.3)	6,798,237 (94.6)	1.15 [0.95-1.39]	1.01 [0.83-1.22]	0.91 [0.76-1.08]
**Age (years) - Median [IIQ]**	4 [2-7]	6 [3-8]			
**Age (years) – n (%)**					
1-4	1,254 (51.8)	2,635,420 (36.7)	1	1	
5-9	935 (38.7)	3,631,695 (50.6)	0.54 [0.49-0.58]	0.74 [0.67-0.81]	
10-12	229 (9.5)	915,171 (12.7)	0.52 [0.45-0.60]	0.77 [0.67-0.90]	
**Sex – n (%)**					
Male	1,387 (57.4)	3,677,321 (51.2)	1	1	
Female	1,031 (42.6)	3,504,965 (48.8)	0.78 [0.71-0.84]	0.85 [0.79-0.93]	
**CMU beneficiaries – n (%)**	666 (27.5)	1,142,353 (15.9)	2.01 [1.83-2.19]	1.65 [1.51-1.81]	
**Region – n (%)**					
Île-de-France	956 (39.5)	1,412,757 (19.7)	1	1	
Northwest	221 (9.1)	1,444,059 (20.1)	0.22 [0.19-0.26]	0.21 [0.18-0.24]	
Northeast	506 (20.9)	1,591,563 (22.2)	0.47 [0.42-0.52]	0.39 [0.35-0.44]	
Southeast	554 (23.0)	1,804,487 (25.1)	0.45 [0.40-0.50]	0.42 [0.38-0.47]	
Southwest	177 (7.3)	905,265 (12.6)	0.28 [0.24-0.33]	0.26 [0.22-0.30]	
Unknown	4 (0.2)	24,155 (0.3)			
**Social deprivation index (quintiles) – n (%)**					
1 (the least deprivation)	556 (23.0)	1,434,539 (20.0)	1	1	
2	451 (18.7)	1,452,496 (20.2)	0.80 [0.70-0.90]	1.09 [0.96-1.24]	
3	411 (17.0)	1,400,985 (19.5)	0.75 [0.66-0.86]	1.13 [0.99-1.29]	
4	414 (17.1)	1,369,381 (19.1)	0.78 [0.68-0.88]	1.16 [1.01-1.32]	
5 (the most deprivation)	561 (23.2)	1,392,622 (19.4)	1.03 [0.92-1.16]	1.33 [1.16-1.51]	
Unknown	25 (1.0)	132,263 (1.8)			

**Exposed to other vaccine – n (%)**	2,375 (98.2)	7,064,563 (98.4)	0.92 [0.68-1.24]		
**Number of previous consultations with a general practitioner or pediatrician– n (%)**					
No consultation	134 (5.5)	559,241 (7.9)	1.07 [0.87-1.30]		1.06 [0.86-1.30]
1-3	325 (13.4)	1,450,992 (20.2)	1		1
4-7	535 (22.1)	1,999,744 (27.8)	1.19 [1.04-1.37]		1.10 [0.96-1.26]
8-12	508 (21.1)	1,560,170 (21.7)	1.45 [1.26-1.67]		1.10 [0.95-1.27]
13 or more	916 (37.9)	1,612,139 (22.4)	2.53 [2.23-2.87]		1.34 [1.16-1.54]
**Previous hospitalizations – n (%)**	538 (22.2)	329,636 (4.6)	5.94 [5.40-6.54]		3.44 [3.09-3.82]
**Comorbidities – n (%)**					
Neurological/Neuromuscular	135 (5.5)	55,597 (0.8)	7.58 [6.37-9.01]		
Respiratory	513 (21.2)	580,671 (8.1)	3.06 [2.77-3.37]		
Cardiovascular	54 (2.2)	21,747 (0.3)	7.52 [5.74-9.85]		
Psychiatric	72 (2.9)	92,163 (1.3)	2.36 [1.86-2.98]	**At least one comorbidities**	2.81 [2.56-3.08]
Metabolic	41 (1.6)	14,656 (0.2)	8.43 [6.19-11.4]		
Gastrointestinal	31 (1.2)	8,666 (0.1)	10.7 [7.53-15.3]		
Renal/Urological	21 (0.8)	13,503 (0.2)	4.65 [3.02-7.15]		
Hematological/Immunological	121 (5.0)	15,152 (0.2)	24.9 [20.7-29.9]		
Genetic/Congenital defect	54 (2.2)	20,298 (0.3)	8.06 [6.15-10.5]		
Cancer	26 (1.1)	5,282 (0.1)	14.7 [10.0-21.7]		
**Intensive Care Unit Admission / death**	132/5				

Supplementary figures

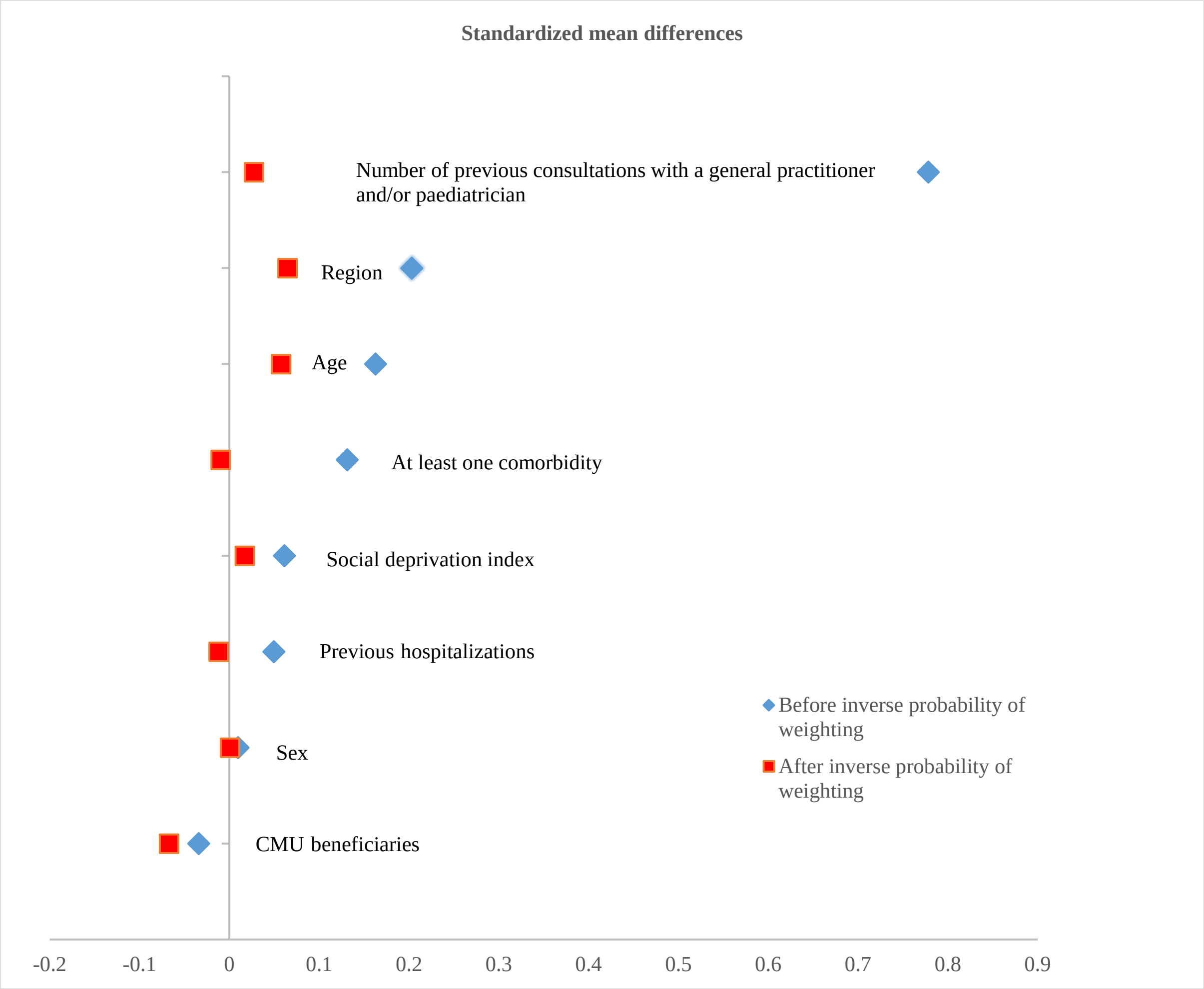

Figure S1. Standardized mean differences before and after inverse probability of weighting.
